# TESTING A CONTEXT-ENRICHED MODEL OF SUCCESSFUL AGING IN MULTIPLE REPRESENTATIVE SAMPLES OF VERY OLD ADULTS

**DOI:** 10.1093/geroni/igae098.0281

**Published:** 2024-12-31

**Authors:** Roman Kaspar, Hans-Werner Wahl

**Affiliations:** Charlotte Fresenius Hochschule, Cologne, Nordrhein-Westfalen, Germany; Heidelberg University, Heidelberg, Baden-Wurttemberg, Germany

## Abstract

Objectively measurable outcomes of successful aging (SA) as used in Rowe and Kahn’s model of SA always operate in multiple contexts that may or may not be perceived as providing opportunity structures for aging well. Context may become most critical in advanced old age. We posit that so far, no empirical test of a contextually enriched model of SA in advanced old age exists. We use multiple representative survey samples of very old individuals, i.e., the D80+ study (study 1; N = 3.233) and the NRW80+ study (study 2; N [baseline] = 1.863; N [wave 2, new independent sample] = 950; N [2-year panel sample] = 912). Study protocols were highly similar across studies and included a multidimensional self-report- based assessment of perceived aging context. Whereas rates of SA according to Rowe and Kahn decreased from 9.1% in those 80-84 years to 0.7% in those 90 years or older, rates for those with many contextually driven opportunity structures at their disposal even if not fulfilling Rowe and Kahn’s criteria were much higher and decreased less steeply from 54.9% (80-84 years) to 44.4% (90 years and older). Also, greater stability of SA across 2 years was observed in supportive contexts. Decontextualizing SA may result in misleadingly low rates of SA in very old adults. Our findings underline the demand to go beyond SA as a process/outcome only at the individual level and aim future policies for aging well much more at strengthening opportunity structures.

